# Brain hemorrhage recurrence, small vessel disease type, and cerebral microbleeds: A meta-analysis

**DOI:** 10.1212/WNL.0000000000004259

**Published:** 2017-08-22

**Authors:** Andreas Charidimou, Toshio Imaizumi, Solene Moulin, Alexandro Biffi, Neshika Samarasekera, Yusuke Yakushiji, Andre Peeters, Yves Vandermeeren, Patrice Laloux, Jean-Claude Baron, Mar Hernandez-Guillamon, Joan Montaner, Barbara Casolla, Simone M. Gregoire, Dong-Wha Kang, Jong S. Kim, H. Naka, Eric E. Smith, Anand Viswanathan, Hans R. Jäger, Rustam Al-Shahi Salman, Steven M. Greenberg, Charlotte Cordonnier, David J. Werring

**Affiliations:** From the Stroke Research Centre (A.C., Y.Y., S.M.G., H.R.J., D.J.W.), Department of Brain Repair and Rehabilitation, UCL Institute of Neurology and The National Hospital for Neurology and Neurosurgery, Queen Square, London, UK; Hemorrhagic Stroke Research Program, Department of Neurology (A.C., A.B., E.E.S., A.V., S.M.G.), Massachusetts General Hospital Stroke Research Center, Harvard Medical School, Boston; Department of Neurosurgery (T.I.), Kushiro City General Hospital, Hokkaido, Japan; Degenerative & Vascular Cognitive Disorders (S.M., B.C., C.C.), Univ Lille, Inserm, CHU Lille, France; Centre for Clinical Brain Sciences (N.S., R.A.-S.S.), University of Edinburgh, UK; Department of Neurology (A.P.), Cliniques Universitaires UCL Saint Luc; Department of Neurology (Y.V., P.L.), CHU Dinant Godinne, Université Catholique de Louvain; Institute of Neuroscience (Y.V., P.L.), Université Catholique de Louvain, Brussels, Belgium; Department of Clinical Neurosciences (J.-C.B.), University of Cambridge, Addenbrooke's Hospital, UK; UMR 894 INSERM-Université Paris 5 (J.-C.B.), Sorbonne Paris Cité, Paris, France; Department of Neurology (M.H.-G., J.M.), Hospital Vall d'Hebron, Vall d'Hebron Research Institute, Universitat Autònoma de Barcelona, Spain; Department of Neurology (D.-W.K., J.S.K.), Asan Medical Center, University of Ulsan College of Medicine, Seoul, South Korea; Department of Neurology (H.N.), Hiroshima Prefectural Hospital, Japan; and Hotchkiss Brain Institute (E.E.S.), University of Calgary, Canada.

## Abstract

**Objective:**

We evaluated recurrent intracerebral hemorrhage (ICH) risk in ICH survivors, stratified by the presence, distribution, and number of cerebral microbleeds (CMBs) on MRI (i.e., the presumed causal underlying small vessel disease and its severity).

**Methods:**

This was a meta-analysis of prospective cohorts following ICH, with blood-sensitive brain MRI soon after ICH. We estimated annualized recurrent symptomatic ICH rates for each study and compared pooled odds ratios (ORs) of recurrent ICH by CMB presence/absence and presumed etiology based on CMB distribution (strictly lobar CMBs related to probable or possible cerebral amyloid angiopathy [CAA] vs non-CAA) and burden (1, 2–4, 5–10, and >10 CMBs), using random effects models.

**Results:**

We pooled data from 10 studies including 1,306 patients: 325 with CAA-related and 981 CAA-unrelated ICH. The annual recurrent ICH risk was higher in CAA-related ICH vs CAA-unrelated ICH (7.4%, 95% confidence interval [CI] 3.2–12.6 vs 1.1%, 95% CI 0.5–1.7 per year, respectively; *p* = 0.01). In CAA-related ICH, multiple baseline CMBs (versus none) were associated with ICH recurrence during follow-up (range 1–3 years): OR 3.1 (95% CI 1.4–6.8; *p* = 0.006), 4.3 (95% CI 1.8–10.3; *p* = 0.001), and 3.4 (95% CI 1.4–8.3; *p* = 0.007) for 2–4, 5–10, and >10 CMBs, respectively. In CAA-unrelated ICH, only >10 CMBs (versus none) were associated with recurrent ICH (OR 5.6, 95% CI 2.1–15; *p* = 0.001). The presence of 1 CMB (versus none) was not associated with recurrent ICH in CAA-related or CAA-unrelated cohorts.

**Conclusions:**

CMB burden and distribution on MRI identify subgroups of ICH survivors with higher ICH recurrence risk, which may help to predict ICH prognosis with relevance for clinical practice and treatment trials.

Spontaneous (nontraumatic) primary intracerebral hemorrhage (ICH) presumed due to cerebral small vessel disease^1^ is a catastrophic form of stroke associated with high morbidity and mortality^2^ and a substantial recurrence risk.^2^ ICH location is associated with the risk of subsequent ICH recurrence,^2^ probably because of the type and severity of the underlying small vessel diseases (microangiopathies), which include arteriolosclerosis, lipohyalinosis, and cerebral amyloid angiopathy (CAA).^3^ The arteriopathy associated with systemic arterial hypertension affects small deep perforating arteries supplying the basal ganglia and deep white matter, resulting in ICH in deep and lobar brain regions.^3^ By contrast, CAA causes progressive vascular deposition of β-amyloid in small cortical and leptomeningeal arterial walls, and is associated with lobar (but not deep) ICH, especially in the elderly.^4–6^ Some studies suggest that CAA-related lobar ICH carries a significantly higher risk for recurrence compared to deep ICH due to hypertensive arteriopathy.^7–10^

Cerebral microbleeds (CMBs), seen on blood-sensitive MRI sequences (e.g., T2*-weighted gradient-recalled echo [T2*-GRE] and susceptibility-weighted imaging), are a radiologic biomarker of cerebral small vessel disease, present in 52% of patients with first-ever ICH and 83% of those with recurrent ICH.^11–13^ Since CMBs sometimes represent blood leakage from hemorrhage-prone small vessels, and their prevalence is higher in recurrent vs first-ever ICH, CMB have been hypothesized to predict increased recurrent ICH risk.^14^ Moreover, the distribution of CMBs can reflect the likely underlying microangiopathy: a strictly lobar distribution (alongside other clinical factors) is highly specific for CAA diagnosis within the Boston criteria.^15,16^ If the risk of ICH recurrence is related to the underlying microangiopathies and their severity, CMB distribution and burden may help to identify patients at high risk of recurrence.

Therefore, we sought published prospective ICH cohorts with MRI (including blood-sensitive sequences) at baseline, to investigate the association of CMB burden and distribution with recurrent ICH in a meta-analysis of aggregate summary-level data, stratified by the presumed underlying microangiopathy (CAA vs CAA-unrelated ICH).

## METHODS

This systematic review and meta-analysis was undertaken using an in-house developed protocol (A.C. and D.J.W.).

### Search strategy, selection criteria, and data extraction.

Two authors (A.C. and D.J.W.) searched PubMed between January 1, 1999, and October 1, 2015, using several combinations of medical subject heading terms and text words: (microbleed* or microhemorrhag* or microhaemorrhag*) and (intracerebral hemorrhage) or (intracerebral haemorrhage) or (brain hemorrhage) or (brain bleed*) and (MRI or MR imaging) and (recurren* or outcome or survival or predict*). Reference lists from all included articles, relevant review articles, and the authors' own files were also searched. Studies were eligible if they included adult patients with spontaneous symptomatic ICH confirmed by imaging and presumed due to sporadic cerebral small vessel disease; had a prospective design, with at least 3 months of follow-up; assessed the risk of recurrent symptomatic spontaneous ICH (main outcome) during follow-up; had data for the presence of CMBs on baseline T2*-GRE MRI; and were published in English. In cases of multiple publications from the same or overlapping cohorts, only the most recent comprehensive results from the report with the largest sample size were used in the analysis. We excluded case-control and cross-sectional studies, case reports, and case series. Two reviewers (A.C. and Y.Y.) determined study eligibility, resolving any disagreements or uncertainties with a third reviewer (D.J.W.) by consensus.

For each study, we extracted data on the country of the study; time period; clinical setting; population size; demographic data (including mean age, sex, and vascular risk factors); use of antithrombotic agents; T2*-GRE MRI parameters; number of participants with at least one CMB at baseline; method and duration of follow-up; and the number of participants with the outcome event of interest. The outcome event of interest was recurrent symptomatic ICH assessed using clear, predefined criteria: namely, symptomatic stroke syndrome associated with neuroimaging evidence of a corresponding ICH. For included cohorts, we sought information from the authors on total person-years of follow-up and outcome events (recurrent ICH) stratified by CMB burden (1, 2–4, 5–10, and >10 CMBs) and distribution (lobar [in the cortex or subcortical areas of the cerebral hemispheres], deep, or both [mixed]).

We classified the study cohorts and subcohorts as CAA-related ICH (including probable and possible CAA, based on the presence of strictly lobar macrobleeds and microbleeds, according to the original Boston criteria) or CAA-unrelated ICH (i.e., including patients with a strictly deep or mixed pattern of CMBs not fulfilling the original Boston criteria,^17^ based on published information and correspondence with authors.

The risk of bias of each included study was assessed against 6 key quality indicators: clearly defined populations, standardized MRI measures, CMB clearly defined per criteria, standardized rating scale used for CMBs rating, standardized definition of outcome (ICH), and completion of follow-up (>90%).

### Statistical analysis.

We estimated recurrent symptomatic ICH (%/year) and corresponding 95% confidence intervals (CIs) for each study from a Poisson regression model and exact Poisson intervals. We calculated pooled rates using the inverse variance method, stratified by study population (CAA-related ICH vs CAA-unrelated ICH). We compared the log (incidence) of recurrent ICH events between these groups using a significance test with the appropriate degrees of freedom.

We meta-analyzed recurrent ICH risks across studies, using a random effects model with DerSimonian-Laird weights,^18^ quantifying the strength of any association using odds ratios (OR) and 95% CI in patients without CMBs vs different CMB burden categories. We analyzed the association between CMBs and ICH recurrence using OR rather than hazard ratios because individual patient data including follow-up time were not available for this meta-analysis. To maximize the power of our analyses, for comparisons with zero events in both groups, we added 0.5 to each group, considered OR = 1, and calculated the SE, logOR, and SE logOR by using the 2-variable input method. We assessed heterogeneity by *I*^2^ and χ^2^ statistics and also visually through inspection of the forest plot and checking for overlapping CIs. We explored publication bias with funnel plots and the Harbord regression tests for funnel plot asymmetry. We stratified all analyses by baseline ICH presumed cause (CAA-related vs CAA-unrelated ICH). We used meta-regression to explore whether certain confounders could have affected our results.

All meta-analyses were performed using Stata 11.2 (StataCorp, College Station, TX). We prepared this report with reference to the Preferred Reporting Items for Systematic Reviews and Meta-Analyses^19^ guidelines.

## RESULTS

**Figure 1:**
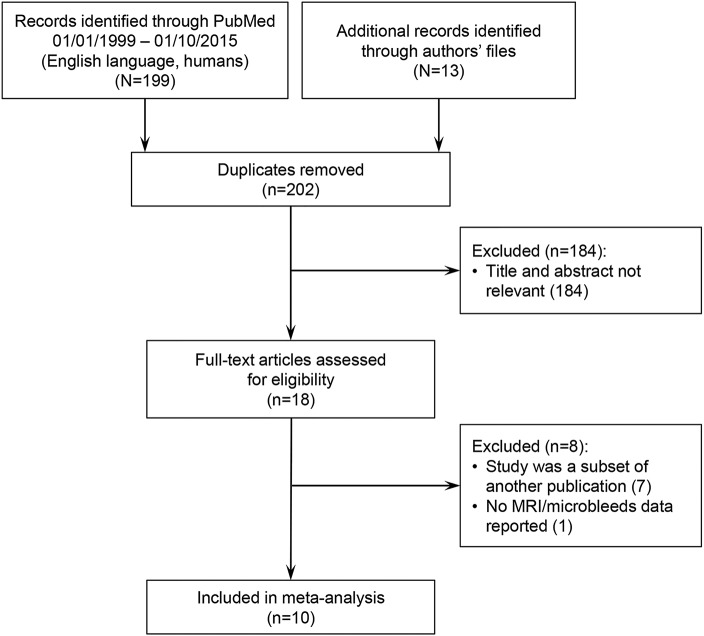
Flow chart of study selection

Ten unique hospital-based studies and 1 population-based study with a total of 1,306 ICH patients met our predefined inclusion criteria (figure 1).^20–29^ The studies comprised 5 CAA-related ICH cohorts (n = 325)^20,23,24,28,29^—3 of which were unselected, and also included CAA-unrelated ICH^24,28,29^—and 5 CAA-unrelated ICH cohorts (n = 981).^21,22,25–27^ Studies had slightly different inception points, variation in the proportion of patients with first-ever vs recurrent ICH, and different prospective and retrospective methods of follow-up (table 1). The risk of bias assessment is summarized in table e-1 at Neurology.org. All studies used T2*-GRE MRI at 1.5T to detect CMBs at baseline, although imaging measures, including echo time and slice thickness, varied slightly. Differences in demographic, clinical, and imaging characteristics between the subgroups with and without CMB are also described in table 1. Overall, compared to CAA-unrelated ICH patients, CAA-related ICH patients in general were older, more often had a prior ICH, and had greater prevalence of white matter hyperintensities (table 1).

**Table 1:**
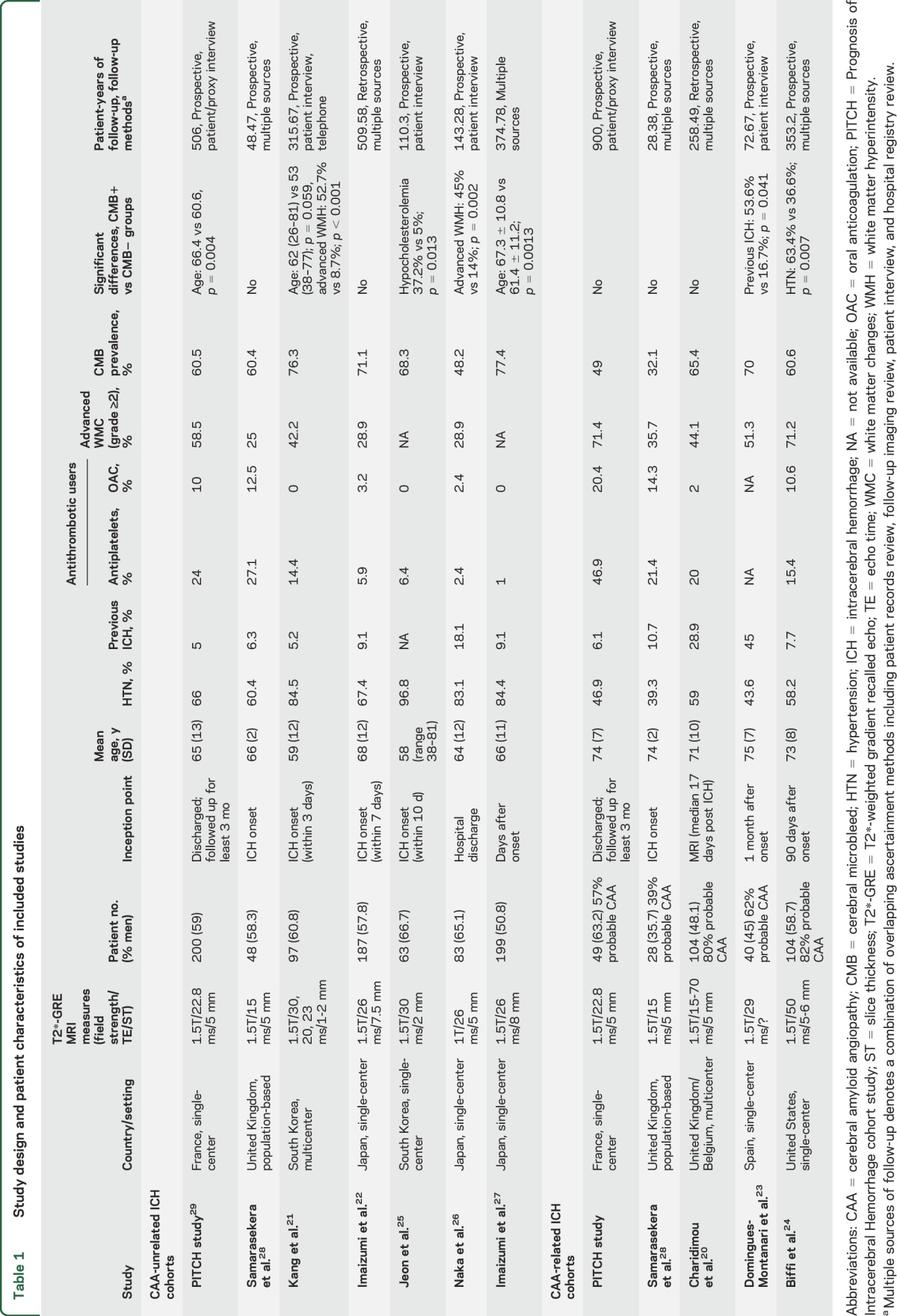
Study design and patient characteristics of included studies

**Figure 2:**
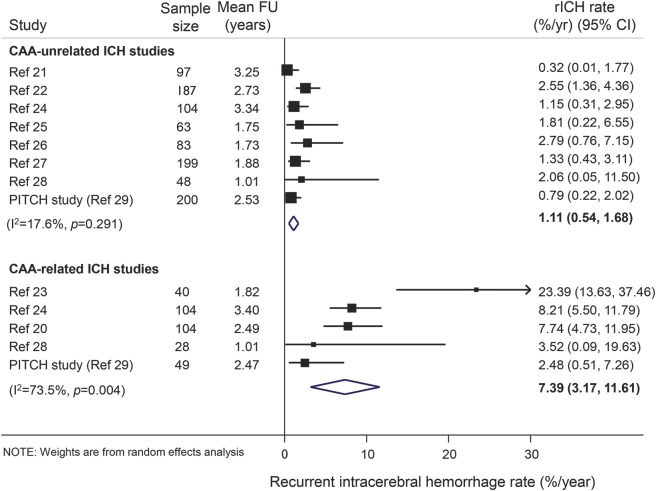
Pooled risks of recurrent symptomatic intracerebral hemorrhage (ICH) during follow-up in included studies Weights are shown by the point estimate area. CAA = cerebral amyloid angiopathy; CI = confidence interval; rICH = recurrent ICH.

Patients with CAA-related ICH had a higher pooled annual risk of recurrent ICH compared to those with CAA-unrelated ICH (7.39%, 95% CI 3.2–12.6 vs 1.1%, 95% CI 0.5–1.7 per year, respectively; *p* = 0.01), but with considerable statistical heterogeneity (figure 2).

**Figure 3:**
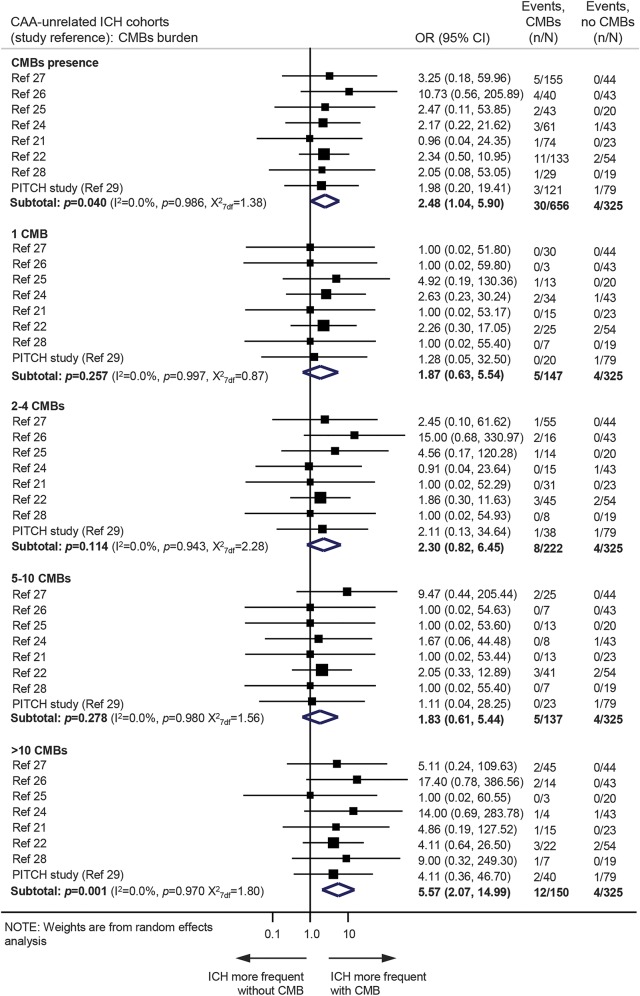
Meta-analysis of the associations between cerebral microbleeds (CMBs) presence or burden and the risk of recurrent symptomatic spontaneous intracerebral hemorrhage (ICH) in cerebral amyloid angiopathy (CAA)–unrelated ICH cohorts Weights are shown by the point estimate area. *I*^2^ is used to test statistical heterogeneity between the subgroup pooled estimates across the different studies. CI = confidence interval; OR = odds ratio.

In the CAA-unrelated ICH cohorts, among patients with CMBs, 30/656 (4.6%, 95% CI 3.1%–6.5%) experienced recurrent ICH, compared to 4/325 (1.2%, 95% CI 0.3%–3.1%) patients without CMBs. The presence of CMBs was associated with an increased risk of recurrent ICH (OR 2.48, 95% CI 1.0–5.9; *p* = 0.04) (figure 3). Although ICH risk seemed to increase with increasing CMB burden, only patients with >10 CMBs had a statistically significant increase in risk compared to patients without CMB (OR 5.6, 95% CI 2.1–15; *p* = 0.001) (figure 3). When we pooled data based on CMBs location, the presence of mixed CMBs (but not strictly lobar or strictly deep) was associated with higher risk of recurrent ICH (data not shown). The results were consistent from study to study (test for heterogeneity *p* > 0.10).

**Figure 4:**
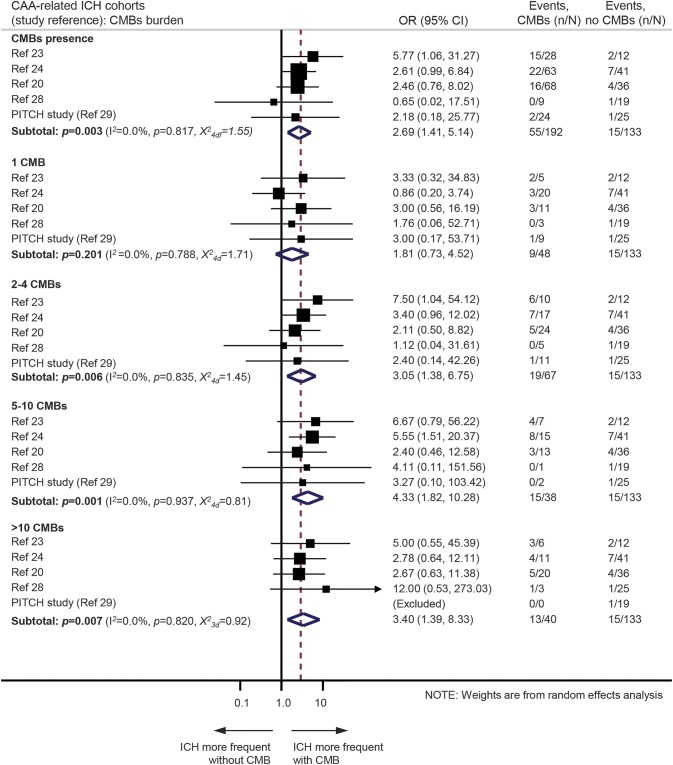
Meta-analysis of the associations between cerebral microbleeds (CMBs) presence or burden and the risk of recurrent spontaneous intracerebral hemorrhage (ICH) in cerebral amyloid angiopathy (CAA)–related ICH cohorts Weights are shown by the point estimate area. *I*^2^ is used to test statistical heterogeneity between the subgroup pooled estimates across the different studies. CI = confidence interval; OR = odds ratio.

In the CAA-related ICH cohorts, 55/192 (28.7%, 95% CI 22.4%–35.6%) patients with CMBs and 15/133 (11.3%, 95% CI 6.5%–17.9%) patients without CMBs had recurrent ICH during follow-up. CMB presence was associated with recurrent ICH risk (OR 2.7, 95% CI 1.4–5.1; *p* = 0.003) (figure 4). The presence of a single CMB was not associated with a higher risk of recurrence compared to CAA patients without any CMBs. However, there was a substantial risk of recurrent ICH with greater CMB burden categories (figure 4).

We used meta-regression to see whether certain confounders could have affected our results. No significant difference was noted in our estimates when we included age, sex, hypertension at baseline, history of previous ICH, white matter hyperintensities, or prior use of antithrombotic medication (antiplatelets or anticoagulants) in the model for our main outcomes. Sensitivity analyses involving sequential removal of each individual study in turn yielded very similar results for all comparisons. Estimation of publication bias via the Egger test and the Begg test returned nonsignificant results (all analyses *p* > 0.20).

## DISCUSSION

In this meta-analysis of 10 cohorts involving more than 1,300 survivors of symptomatic spontaneous ICH who underwent blood-sensitive MRI, pooled estimates demonstrated a 7-fold increase in the risk of recurrent ICH after CAA-related ICH compared to CAA-unrelated ICH. We found a consistent association between CMB presence at baseline and future ICH recurrence, but the strength of the association of CMBs and the magnitude of recurrent ICH risk differed according to underlying microangiopathy.

Our finding of high ICH risk in probable CAA-related ICH is in line with most previous studies^10,30–32^ and systematic reviews^2,7^ that found ICH recurrence is more common following lobar ICH (whether related to CAA or not) than nonlobar ICH. However, few previous studies of ICH prognosis have used MRI to systematically phenotype the likely underlying small vessel arteriopathy.^2,7^ MRI has emerged as the most useful noninvasive method to diagnose the microangiopathies associated with spontaneous ICH. Our data suggest that MRI may also be valuable in assessing prognosis.

The differential stroke risks in ICH survivors have implications for prognosis and secondary prevention decisions, especially antithrombotic treatment, an increasingly common clinical dilemma.^33,34^ Our data suggest that MRI could identify a specific subgroup of patients with ICH at highest risk for further hemorrhagic events. A decision analysis based on a 69-year-old survivor of a lobar CAA-related ICH and newly diagnosed nonvalvular atrial fibrillation suggested that such patients should not be anticoagulated with warfarin across the spectrum of thromboembolic and hemorrhagic risks.^35^ However, the value of MRI in stratifying patients according to their risks for recurrent ICH and ischemic events needs to be tested in randomized controlled trials.

We did not find a significantly elevated risk for recurrent ICH associated with the presence of a single CMB relative to the absence of CMB in any of the cohorts. The pathophysiologic significance of a single CMB is unclear as it might indicate a less severe microangiopathy than in patients with multiple CMBs. Furthermore, rating of a single CMB may be less reliable than multiple CMBs. It is important to note that all the included studies used standard clinical T2*-GRE MRI and on 1.5T systems. The use of higher magnetic field strength and of susceptibility-weighted imaging increase sensitivity to CMB detection,^36^ and it is therefore conceivable that these techniques might have detected additional CMBs, affecting the preselected cutoffs used for microbleeds grouping.

We have confirmed previous reports suggesting that lobar CMB burden at baseline predicts recurrent ICH on clinical follow-up,^24^ making CMBs promising prognostic biomarkers in CAA clinical practice and research studies. In previous studies, the occurrence of CAA-related recurrent lobar ICH was lower than the rate of new CMB development.^37^ Different vasculopathic features and environmental exposures may determine whether a vessel rupture will result in a CMB or a larger macrobleed. In non-CAA-related ICH cohorts, our results indicate that more than 10 CMBs predict recurrent ICH risk.

Our meta-analysis has limitations. Some studies had a small sample size, variable follow-up duration and methods, and few outcome events, leading to wide CIs around risk estimates. In these studies, inception points and methods of outcome assessment varied. Imaging measures, for example echo time, varied across studies, potentially affecting the detection of CMBs, although the consistent prevalence does not suggest measurement error, especially since all cohorts were scanned at 1.5T using T2*-GRE, probably making variation minimal. Furthermore, the cohorts included may have been subject to selection bias (since not all ICH patients can have MRI), so our findings can only be generalized to ICH survivors who undergo T2*-GRE MRI in clinical practice. Since our meta-analysis included group-level data, we were unable to test the influence of CMB count on a continuous scale; instead, we have used prespecified clinically relevant CMB burden groups. Ischemic stroke was not specifically identified or reported by all of the cohorts, making these data unreliable. Confounding may explain some of the difference between the risks of recurrent ICH in CAA-related and CAA-unrelated cohorts, since the CAA-related cohorts were older, had a higher frequency of prior ICH, and had more white matter disease (table 1). However, all studies showed a consistent direction of association between CMBs and recurrent ICH, even when adjusted for these potential confounders. Finally, we compared groups using ORs rather than hazard ratios, based on numbers of events in each group during variable periods of follow-up for individual patients.

Despite our best efforts, including meta-regression analyses, there is likely still some residual confounding of our estimates. For example, ethnicity (Asian vs non-Asian cohorts) may contribute to heterogeneity, considering that Asian populations have a higher overall risk of ICH and different vascular risk factors. Of note, all CAA cohorts included mainly non-Asian patients, in line with CAA accounting for a smaller proportion of ICH due to the higher prevalence of hypertensive arteriopathy in Asian populations.^38^ Hence heterogeneity could be more relevant in the non-CAA ICH cohorts, which requires further study. Moreover, factors other than CMB burden may also play a role in ICH recurrence risk, including other hemorrhagic markers of small vessel disease (cortical superficial siderosis in particular^39^), blood pressure variability and control,^40^ and antithrombotic drug use. The interaction between CMB counts and these factors on the risk of recurrent ICH are also of interest, but these data were not available for our analysis; this is an active area of research. In CAA-related ICH, patients with 5–10 CMBs had a higher OR (4.3, 95% CI 1.8–10.3; *p* = 0.001) for ICH recurrence than patients with >10 CMBs (3.4, 95% CI 1.4–8.3; *p* = 0.007), which might be due to a ceiling effect for >5 CMBs. Well-designed multicenter prospective pooled studies of individual patient data with systematic and standardized follow-up are required to fully explore the influence and interactions of clinical variables, MRI markers, and vascular risk factor treatment strategies.

Spontaneous ICH originates from a variety of cerebral microangiopathies with potentially distinct future stroke risks (including recurrent ICH and ischemic stroke). Our findings suggest that using MRI to determine the presumed microangiopathies underlying spontaneous ICH can improve estimation of future recurrent ICH risk. This is important to inform patients and caregivers, plan clinical services, and design clinical trials. Our data suggest that the presence and burden of CMBs on blood-sensitive MRI sequences are important for stratifying patients more prone to recurrent ICH, but their role in identifying patients at higher risk for ischemic stroke could not be addressed. Whether the balance of risk of recurrent ICH and ischemic stroke after ICH changes over time, and how antithrombotic drugs (antiplatelet and anticoagulant agents) influence this, remain important questions for randomized controlled trials, such as the ongoing RESTART (registered ISRCTN71907627) and APACHE-AF (EudraCT number: 2014-000112-33) trials testing whether a policy of starting antiplatelet or anticoagulant drugs results in a beneficial net reduction of serious vascular events compared with a policy of avoiding antithrombotic drugs.

## Supplementary Material

Data Supplement

## GLOSSARY

CAA: cerebral amyloid angiopathy
CI: confidence interval
CMB: cerebral microbleed
GRE: gradient-recalled echo
ICH: intracerebral hemorrhage
OR: odds ratio

## References

[1] Wardlaw JM, Smith EE, Biessels GJ, et al. Neuroimaging standards for research into small vessel disease and its contribution to ageing and neurodegeneration. Lancet Neurol 2013;12:822–838. 23867200 10.1016/S1474-4422(13)70124-8 PMC3714437

[2] Poon MT, Fonville AF, Al-Shahi Salman R. Long-term prognosis after intracerebral haemorrhage: systematic review and meta-analysis. J Neurol Neurosurg Psychiatry 2014;85:660–667. 24262916 10.1136/jnnp-2013-306476

[3] Pantoni L. Cerebral small vessel disease: from pathogenesis and clinical characteristics to therapeutic challenges. Lancet Neurol 2010;9:689–701. 20610345 10.1016/S1474-4422(10)70104-6

[4] Charidimou A, Gang Q, Werring DJ. Sporadic cerebral amyloid angiopathy revisited: recent insights into pathophysiology and clinical spectrum. J Neurol Neurosurg Psychiatry 2012;83:124–137. 22056963 10.1136/jnnp-2011-301308

[5] Viswanathan A, Greenberg SM. Cerebral amyloid angiopathy in the elderly. Ann Neurol 2011;70:871–880. 22190361 10.1002/ana.22516 PMC4004372

[6] Samarasekera N, Smith C, Al-Shahi Salman R. The association between cerebral amyloid angiopathy and intracerebral haemorrhage: systematic review and meta-analysis. J Neurol Neurosurg Psychiatry 2012;83:275–281. 22056966 10.1136/jnnp-2011-300371

[7] Bailey RD, Hart RG, Benavente O, Pearce LA. Recurrent brain hemorrhage is more frequent than ischemic stroke after intracranial hemorrhage. Neurology 2001;56:773–777. 11274313 10.1212/wnl.56.6.773

[8] Viswanathan A, Rakich SM, Engel C, et al. Antiplatelet use after intracerebral hemorrhage. Neurology 2006;66:206–209. 16434655 10.1212/01.wnl.0000194267.09060.77

[9] Weimar C, Benemann J, Terborg C, Walter U, Weber R, Diener HC. Recurrent stroke after lobar and deep intracerebral hemorrhage: a hospital-based cohort study. Cerebrovasc Dis 2011;32:283–288. 21893981 10.1159/000330643

[10] Hill MD, Silver FL, Austin PC, Tu JV. Rate of stroke recurrence in patients with primary intracerebral hemorrhage. Stroke 2000;31:123–127. 10625726 10.1161/01.str.31.1.123

[11] Greenberg SM, Vernooij MW, Cordonnier C, et al. Cerebral microbleeds: a guide to detection and interpretation. Lancet Neurol 2009;8:165–174. 19161908 10.1016/S1474-4422(09)70013-4 PMC3414436

[12] Lee SH, Ryu WS, Roh JK. Cerebral microbleeds are a risk factor for warfarin-related intracerebral hemorrhage. Neurology 2009;72:171–176. 19139370 10.1212/01.wnl.0000339060.11702.dd

[13] Cordonnier C, Al-Shahi Salman R, Wardlaw J. Spontaneous brain microbleeds: systematic review, subgroup analyses and standards for study design and reporting. Brain 2007;130:1988–2003. 17322562 10.1093/brain/awl387

[14] Charidimou A, Werring DJ. Cerebral microbleeds as a predictor of macrobleeds: what is the evidence? Int J Stroke 2014;9:457–459. 24798040 10.1111/ijs.12280

[15] Lee SH, Bae HJ, Kwon SJ, et al. Cerebral microbleeds are regionally associated with intracerebral hemorrhage. Neurology 2004;62:72–76. 14718700 10.1212/01.wnl.0000101463.50798.0d

[16] Linn J, Halpin A, Demaerel P, et al. Prevalence of superficial siderosis in patients with cerebral amyloid angiopathy. Neurology 2010;74:1346–1350. 20421578 10.1212/WNL.0b013e3181dad605 PMC2875936

[17] Knudsen KA, Rosand J, Karluk D, Greenberg SM. Clinical diagnosis of cerebral amyloid angiopathy: validation of the Boston criteria. Neurology 2001;56:537–539. 11222803 10.1212/wnl.56.4.537

[18] DerSimonian R, Laird N. Meta-analysis in clinical trials. Control Clin Trials 1986;7:177–188. 3802833 10.1016/0197-2456(86)90046-2

[19] Moher D, Liberati A, Tetzlaff J, Altman DG. Preferred reporting items for systematic reviews and meta-analyses: the PRISMA statement. BMJ 2009;339:b2535. 19622551 10.1136/bmj.b2535 PMC2714657

[20] Charidimou A, Peeters AP, Jager R, et al. Cortical superficial siderosis and intracerebral hemorrhage risk in cerebral amyloid angiopathy. Neurology 2013;81:1666–1673. 24107862 10.1212/01.wnl.0000435298.80023.7a PMC3812101

[21] Kang DW, Han MK, Kim HJ, et al. New ischemic lesions coexisting with acute intracerebral hemorrhage. Neurology 2012;79:848–855. 22843271 10.1212/WNL.0b013e3182648a79

[22] Imaizumi T, Inamura S, Kohama I, et al. Antithrombotic drug uses and deep intracerebral hemorrhages in stroke patients with deep cerebral microbleeds. J Stroke Cerebrovasc Dis 2013;22:869–875. 22959109 10.1016/j.jstrokecerebrovasdis.2012.08.003

[23] Domingues-Montanari S, Hernandez-Guillamon M, Fernandez-Cadenas I, et al. ACE variants and risk of intracerebral hemorrhage recurrence in amyloid angiopathy. Neurobiol Aging 2011;32:551 e13–551.e22. 10.1016/j.neurobiolaging.2010.01.019 20381197

[24] Biffi A, Halpin A, Towfighi A, et al. Aspirin and recurrent intracerebral hemorrhage in cerebral amyloid angiopathy. Neurology 2010;75:693–698. 20733144 10.1212/WNL.0b013e3181eee40f PMC2931649

[25] Jeon SB, Kang DW, Cho AH, et al. Initial microbleeds at MR imaging can predict recurrent intracerebral hemorrhage. J Neurol 2007;254:508–512. 17401517 10.1007/s00415-006-0406-6

[26] Naka H, Nomura E, Takahashi T, et al. Combinations of the presence or absence of cerebral microbleeds and advanced white matter hyperintensity as predictors of subsequent stroke types. AJNR Am J Neuroradiol 2006;27:830–835. 16611773 PMC8133980

[27] Imaizumi T, Horita Y, Hashimoto Y, Niwa J. Dotlike hemosiderin spots on T2*-weighted magnetic resonance imaging as a predictor of stroke recurrence: a prospective study. J Neurosurg 2004;101:915–920. 15597750 10.3171/jns.2004.101.6.0915

[28] Samarasekera N, Fonville A, Lerpiniere C, et al. Influence of intracerebral hemorrhage location on incidence, characteristics, and outcome: population-based study. Stroke 2015;46:361–368. 25586833 10.1161/STROKEAHA.114.007953

[29] Pasquini M, Benedictus MR, Boulouis G, Rossi C, Dequatre-Ponchelle N, Cordonnier C. Incident cerebral microbleeds in a cohort of intracerebral hemorrhage. Stroke 2016;47:689–694. 26839348 10.1161/STROKEAHA.115.011843

[30] Passero S, Burgalassi L, D'Andrea P, Battistini N. Recurrence of bleeding in patients with primary intracerebral hemorrhage. Stroke 1995;26:1189–1192. 7604411 10.1161/01.str.26.7.1189

[31] Yen CC, Lo YK, Li JY, Lin YT, Lin CH, Gau YY. Recurrent primary intracerebral hemorrhage: a hospital based study. Acta Neurol Taiwan 2007;16:74–80. 17685130

[32] Hanger HC, Wilkinson TJ, Fayez-Iskander N, Sainsbury R. The risk of recurrent stroke after intracerebral haemorrhage. J Neurol Neurosurg Psychiatry 2007;78:836–840. 17220294 10.1136/jnnp.2006.106500 PMC2117741

[33] Charidimou A, Werring DJ. The dilemma of atrial fibrillation in intracerebral haemorrhage: how to balance the risks of ischaemia and bleeding. Eur J Neurol 2014;21:549–551. 24010578 10.1111/ene.12258

[34] Horstmann S, Rizos T, Jenetzky E, Gumbinger C, Hacke W, Veltkamp R. Prevalence of atrial fibrillation in intracerebral hemorrhage. Eur J Neurol 2014;21:570–576. 23906054 10.1111/ene.12215

[35] Eckman MH, Rosand J, Knudsen KA, Singer DE, Greenberg SM. Can patients be anticoagulated after intracerebral hemorrhage? A decision analysis. Stroke 2003;34:1710–1716. 12805495 10.1161/01.STR.0000078311.18928.16

[36] Charidimou A, Krishnan A, Werring DJ, Rolf Jager H. Cerebral microbleeds: a guide to detection and clinical relevance in different disease settings. Neuroradiology 2013;55:655–674. 23708941 10.1007/s00234-013-1175-4

[37] Greenberg SM, Eng JA, Ning M, Smith EE, Rosand J. Hemorrhage burden predicts recurrent intracerebral hemorrhage after lobar hemorrhage. Stroke 2004;35:1415–1420. 15073385 10.1161/01.STR.0000126807.69758.0e

[38] Chen YW, Lee MJ, Smith EE. Cerebral amyloid angiopathy in East and West. Int J Stroke 2010;5:403–411. 20854625 10.1111/j.1747-4949.2010.00466.x

[39] Charidimou A, Linn J, Vernooij MW, et al. Cortical superficial siderosis: detection and clinical significance in cerebral amyloid angiopathy and related conditions. Brain 2015;138:2126–2139. 26115675 10.1093/brain/awv162

[40] Biffi A, Anderson CD, Battey TW, et al. Association between blood pressure control and risk of recurrent intracerebral hemorrhage. JAMA 2015;314:904–912. 26325559 10.1001/jama.2015.10082 PMC4737594

