# Supplementary material for: Brain hemorrhage recurrence, small vessel disease type, and cerebral microbleeds: A meta-analysis
Source: Neurology. 2017 Aug 22;89(8):820–9. doi: 10.1212/WNL.0000000000004259 (PMC5580863; doi:10.1212/WNL.0000000000004259)
Supplement: Data Supplement [file supp_89_8_820__index.html]

Brain hemorrhage recurrence, small vessel disease type, and cerebral microbleeds — Data Supplement 

# Brain hemorrhage recurrence, small vessel disease type, and cerebral microbleeds

## Data Supplement

**Neurology® data supplements are not copyedited before publication. Published editorials and translations have been copyedited.  
 © 2017 American Academy of Neurology.  
  
 Files in this Data Supplement:**

- Table e-1 - PDF
