# Supplementary material for: Brain hemorrhage recurrence, small vessel disease type, and cerebral microbleeds: A meta-analysis
Source: Neurology. 2017 Aug 22;89(8):820–9. doi: 10.1212/WNL.0000000000004259 (PMC5580863; doi:10.1212/WNL.0000000000004259)
Supplement: Data Supplement [file supp_WNL.0000000000004259_Table_e-1.pdf]

Table e-1: Risk of bias assessment based on key quality indicators for the included studies.

Study (primary author or name)	Clearly defined populations	Standardised MRI parameters	CMB clearly defined per criteria	Standardised rating scale	Standardised definition of outcome (ICH)	Completion of follow up (>90%)	Number of quality indicators
PITCH study	+	+	+	+	+	+	6/6
Samarasekeraet all. 2015	+	+	+	+	+	+	6/6
Kang *et al* 2012	+	+	+	+	+	+	6/6
Imaizumi *et al* 2012	-	+	+	+	+	+	5/6
Jeon *et al* 2007	+	+	+	+	+	-	5/6
Naka *et al* 2006	+	+	+	+	+	+	6/6
Imaizumi *et al* 2004	-	+	+	+	+	+	5/6
Charidimou *et al* 2013	+	+	+	+	+	+	6/6
Domingues-Montanari *et al* 2011	+	+	+	+	+	+	6/6
Biffi *et al* 2010	+	+	+	+	+	+	6/6

+ = present; - = absent; ? = unavailable. CMB – cerebral microbleed; ICH-intracerebral hemorrhage
